# Supplementary material for: The SWI/SNF chromatin remodelling complex is required for maintenance of lineage specific enhancers
Source: Nat Commun. 2017 Mar 6;8:14648. doi: 10.1038/ncomms14648 (PMC5343482; doi:10.1038/ncomms14648)
Supplement: Supplementary Information — Supplementary Figures and Supplementary Table [file ncomms14648-s1.pdf]

Condition	N_t	N_p	N_t/N_p
wild-type	22901	23806	0.96
$Smarca4^{fl/fl}$	11548	9694	1.19
$Smarcb1^{fl/fl}$	15275	20980	0.73

Supplementary Table 1: Number of peaks with $IDR < 0.01$ for true replicates (N_t) and pseudoreplicates(N_p). To obtain IDR values, we have called point binding sites for H3K27ac samples (*find.binding.positions*), with a loose threshold of $FDR < 0.05$. Peaks from two replicates were considered to be the same peaks if they were within 90bp of each other. Peak signal values, y, from the two replicates were used to determine IDR using the *est.IDR* function from R package IDR. If a peak in one sample did not match any peaks in the replicate sample, a signal signal value of $y = 0$ was assigned in the replicate. We have also performed an IDR comparison between two pseudoreplicates generated by randomly partitioning the reads from the two replicates. ENCODE guidelines require that the number of peaks with $IDR < 0.01$ for true replicates (N_t) should be more than 50% of the number of peaks for pseudoreplicates (N_p)

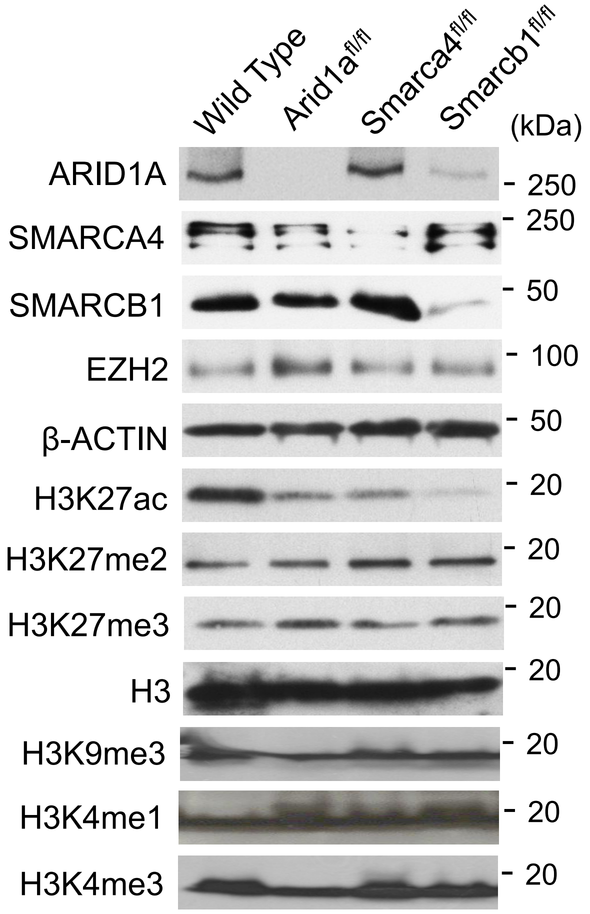

Supplementary Figure 1: Western blots of selected factors, SWI/SNF subunits, histones, and histone modifications for wild type and conditional knockout MEFs (*Arid1a*, *Smarca4*, or *Smarcb1*).

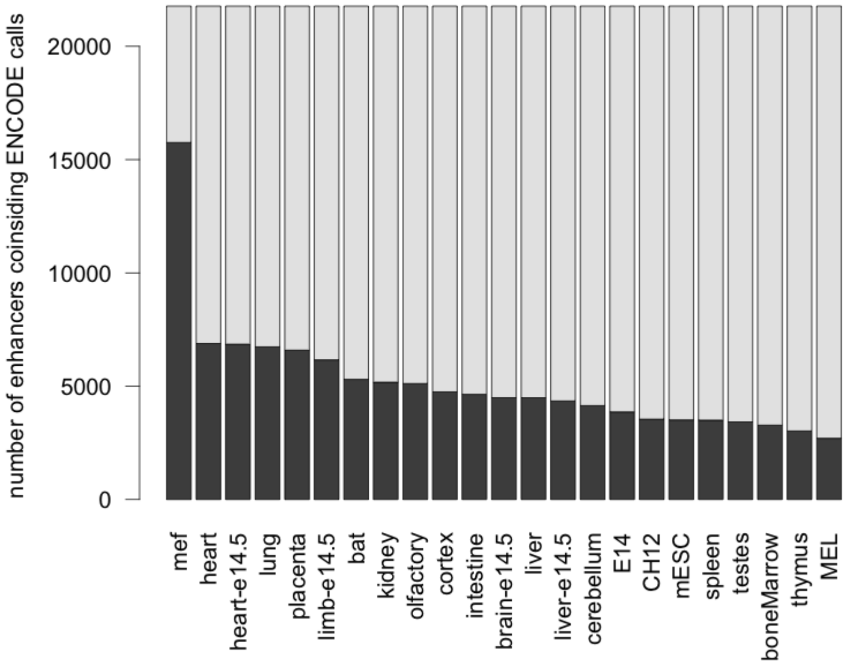

Supplementary Figure 2: Number of enhancers identified in this work that overlap enhancer calls in different cell types by mouse ENCODE consortium. The biggest overlap is seen with the matching cell type.

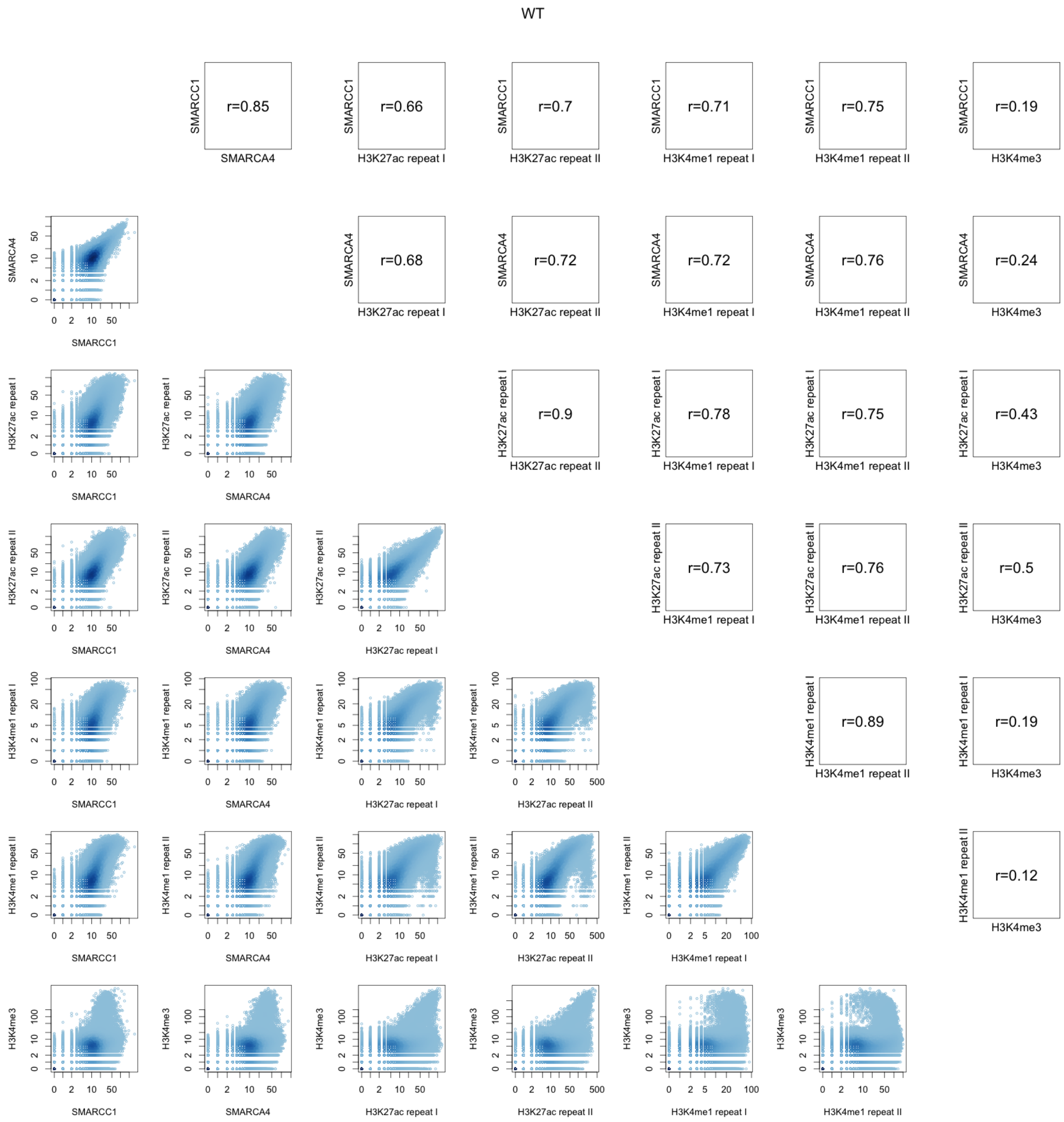

Supplementary Figure 3a: Genomewide correlation of ChIP-seq signal in 1kb bins between different experiments in wild-type. A high degree of correlation is seen for replicates of H3K27ac and H3K4me1. Furthermore the correlation between the two SWI/SNF subunits, SMARCA4 and SMARCC1, is at a similar to the levels of correlation between the replicate histone mark experiments.

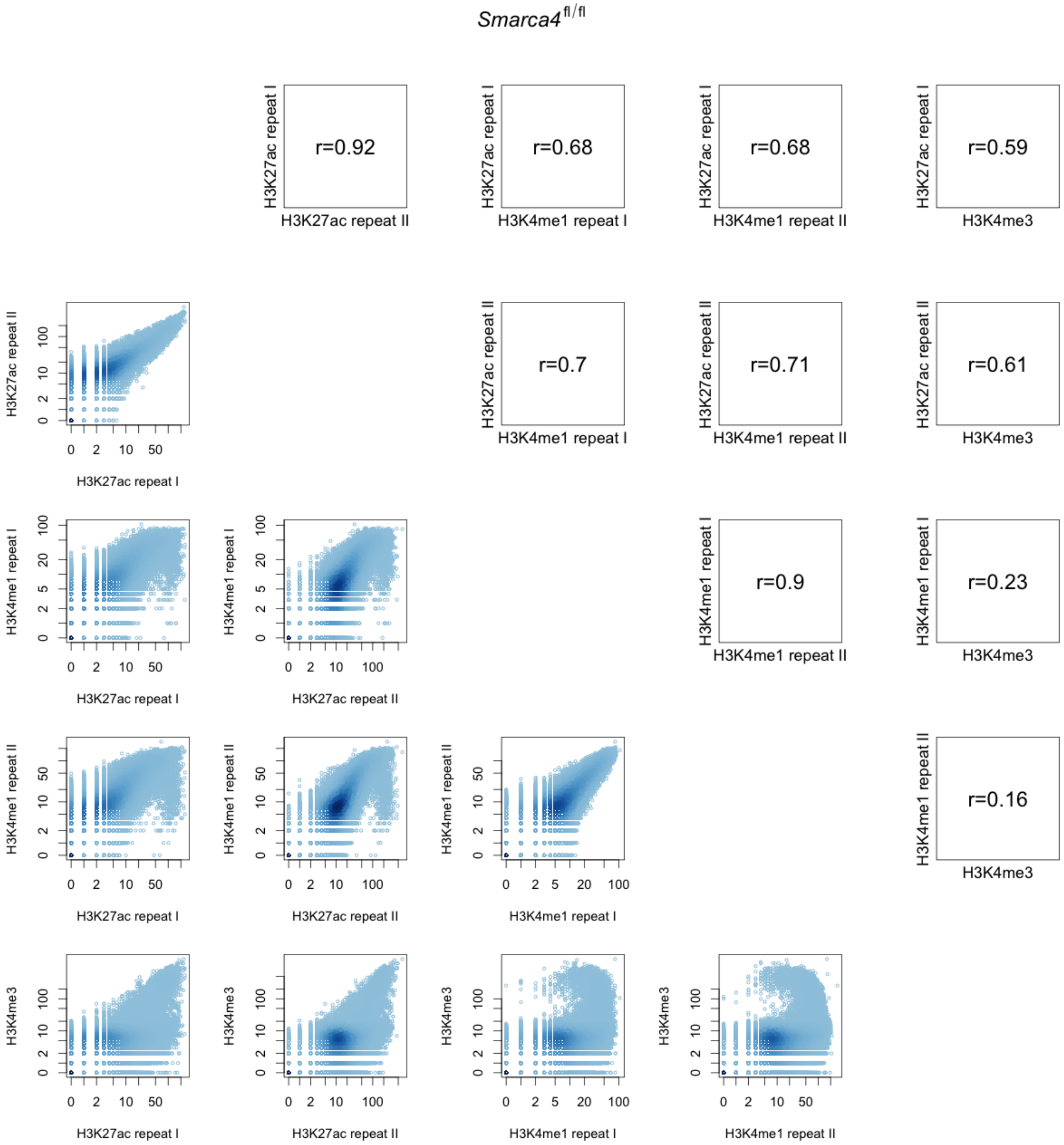

Supplementary Figure 3b: Same as a, in *Smarca4*-deficient cells. SMARCA4 and SMARCC1 ChIP-seq experiments did not pass quality controls in *Smarca4*-deficient samples.

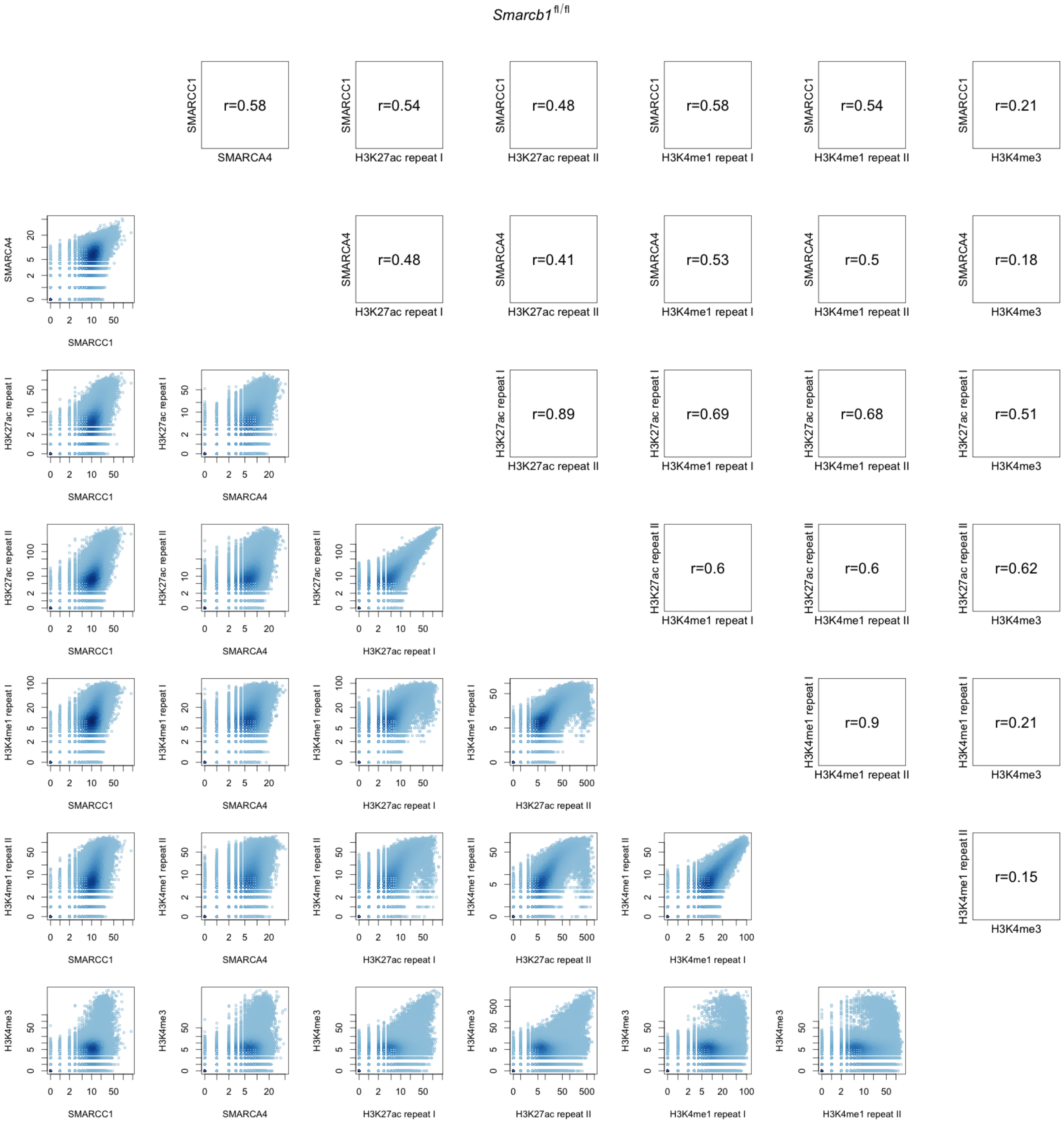

Supplementary Figure 3c: Same as a, in *Smarcb1*-deficient cells. SMARCA4 and SMARCC1 are less enriched and therefore appear less correlated genome-wide.

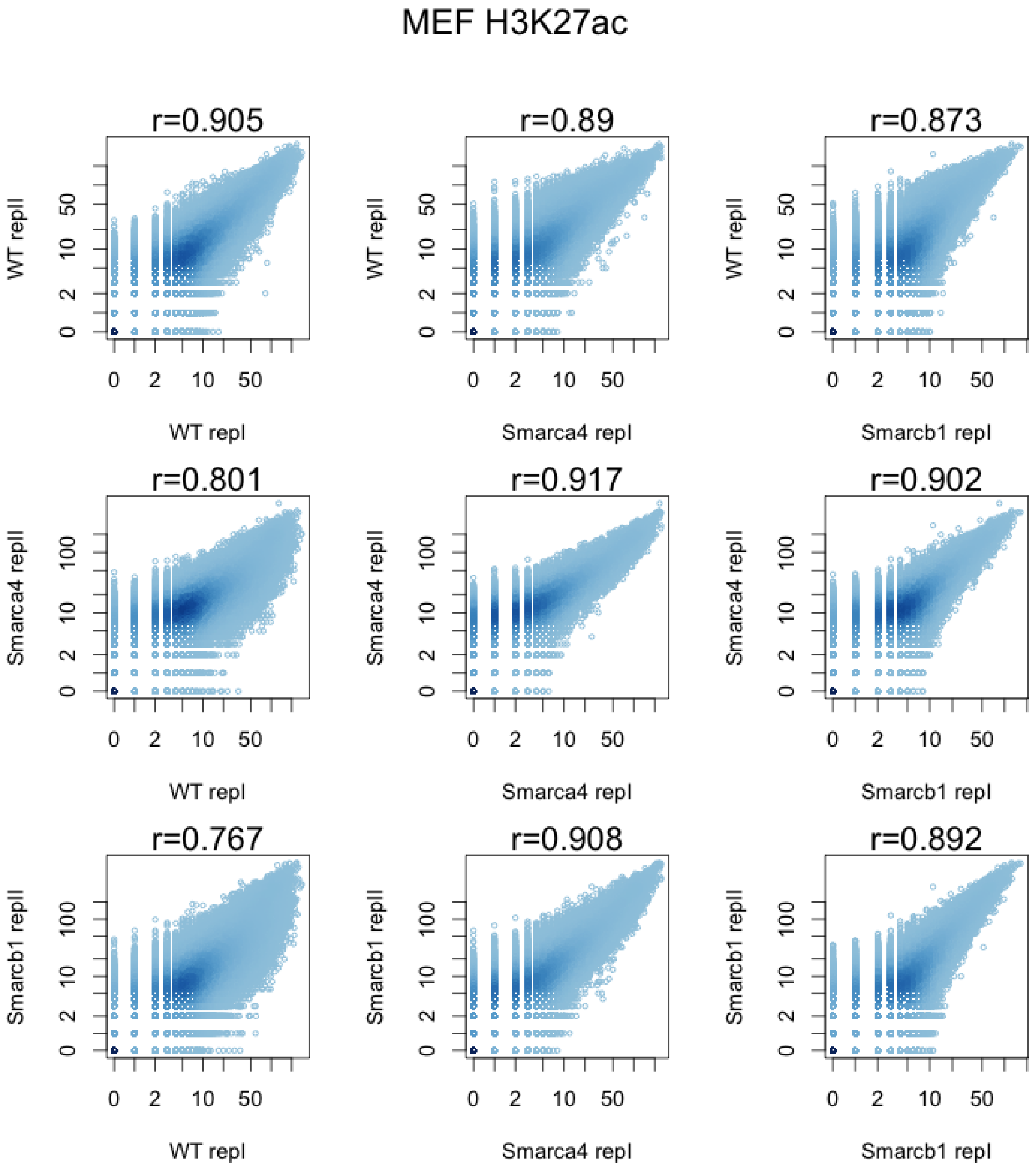

Supplementary Figure 3d: Genomewide correlation of H3K27ac ChIP-seq signal in 1kb bins, showing replicate II vs replicate I, across different conditions.

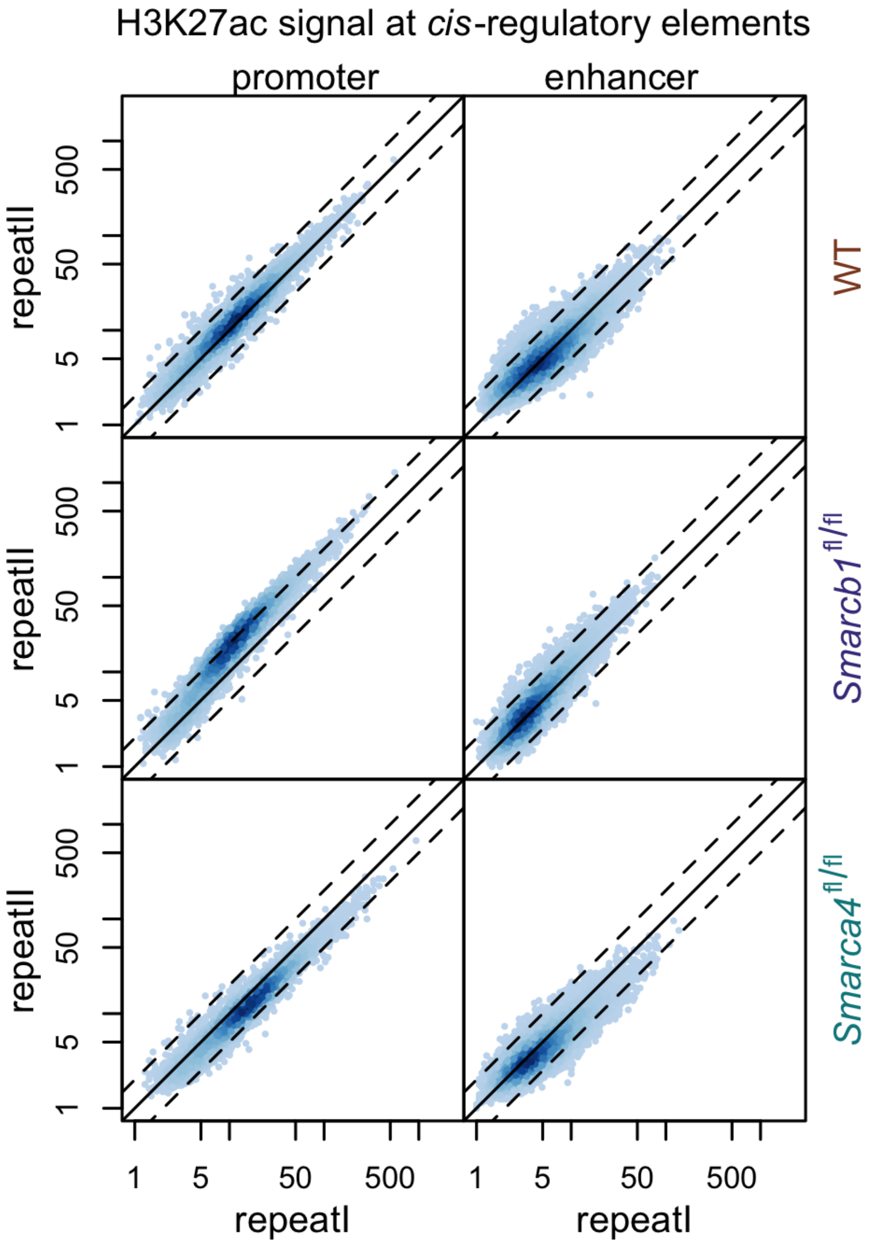

Supplementary Figure 4: Library-size normalized H3K27ac signal at promoters and enhancers in repeat II vs repeat I, before additional normalization. Solid lines show y=x and the dashed lines show 2-fold difference.

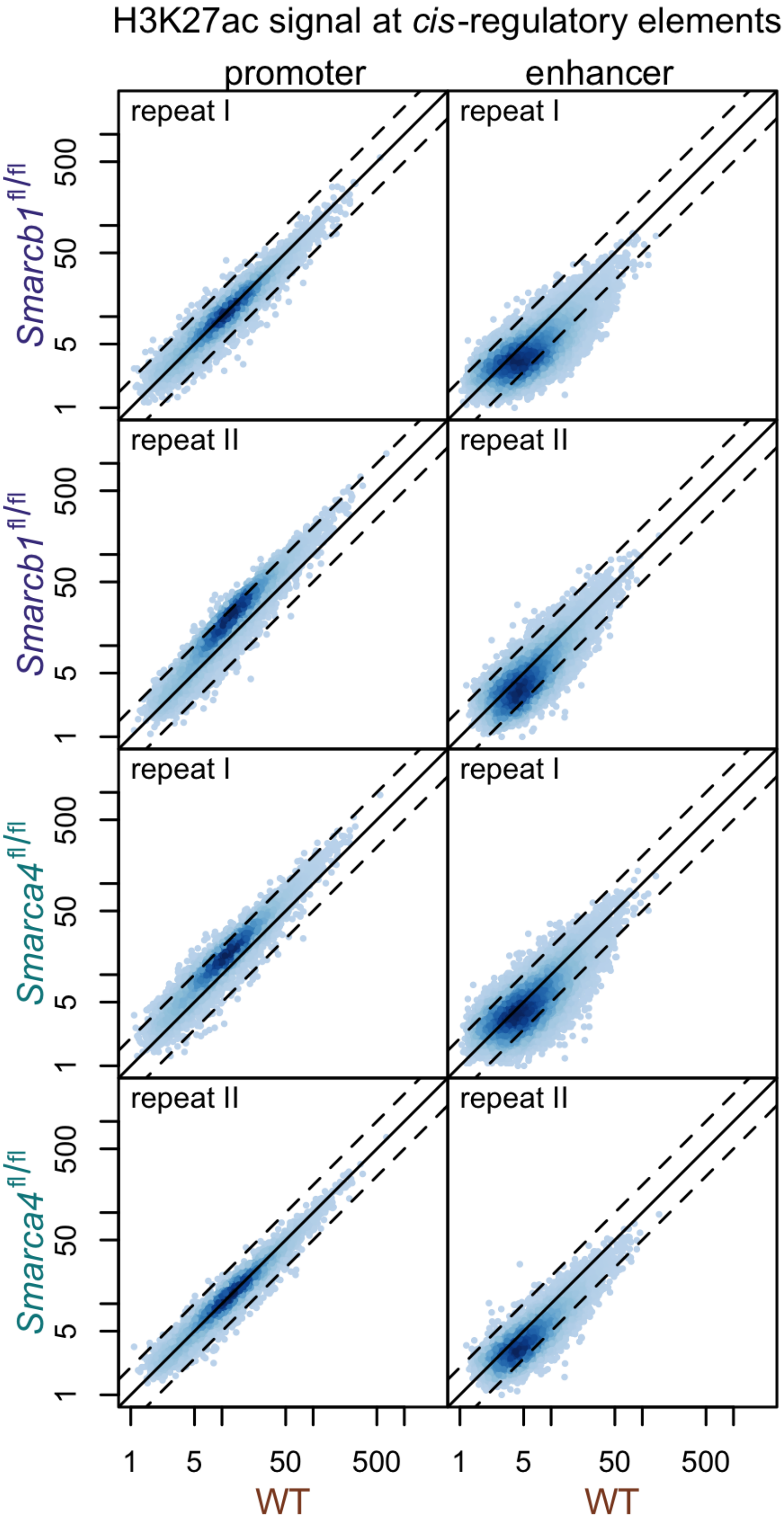

Supplementary Figure 5: Library-size normalized H3K27ac signal at promoters and enhancers in *Smarcb1*-deficient or *Smarca4*-deficient vs. wild-type MEFs, before additional normalization or averaging of replicate experiments. Solid lines show y=x and the dashed lines show 2-fold difference.

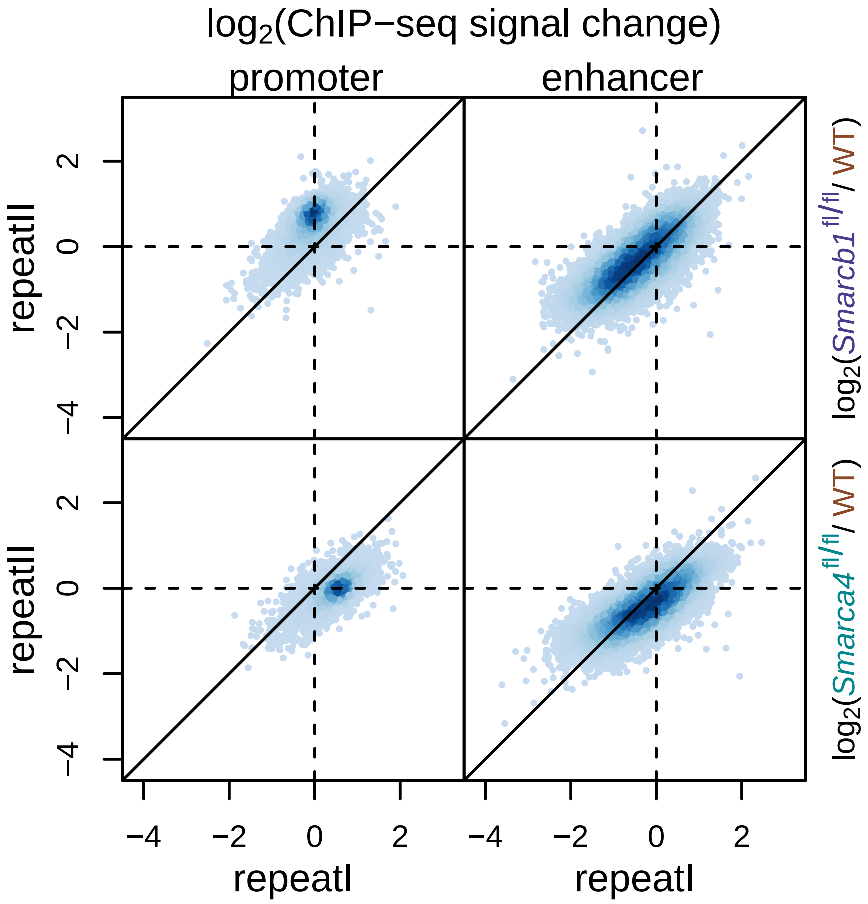

Supplementary Figure 6: Log-fold change of H3K27ac signal at promoters and enhancers in *Smarca4*-deficient or *Smarcb1*-deficient relative to wild-type MEFs, based on library size normalization. The ratios obtained in the second replicate set are plotted against the ratios in the first replicate set.

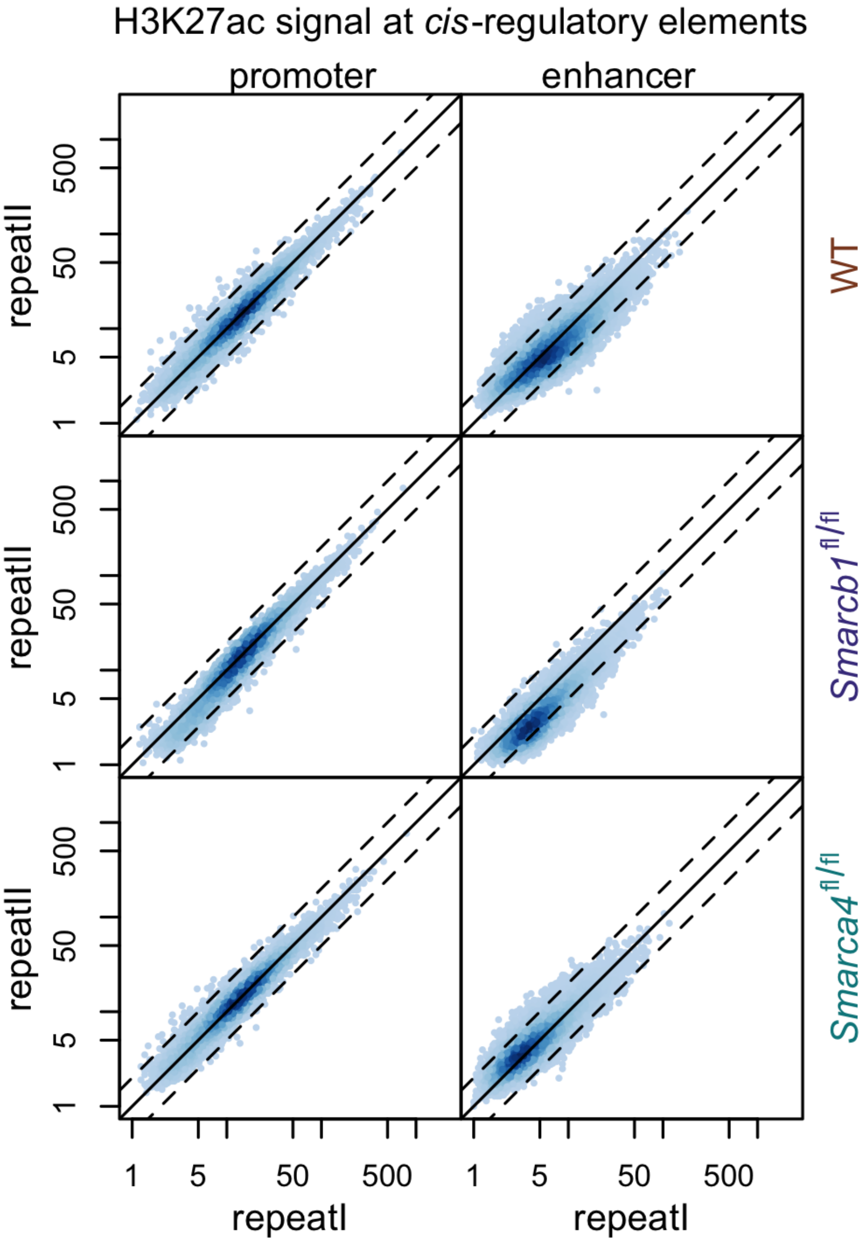

Supplementary Figure 7a: H3K27ac signal at promoters and enhancers in repeat II vs repeat I, after applying a multiplicative correction to account for IP pull-down efficiency. Solid lines show y=x and the dashed lines show 2-fold difference. Compare to Supplementary Figure 4.

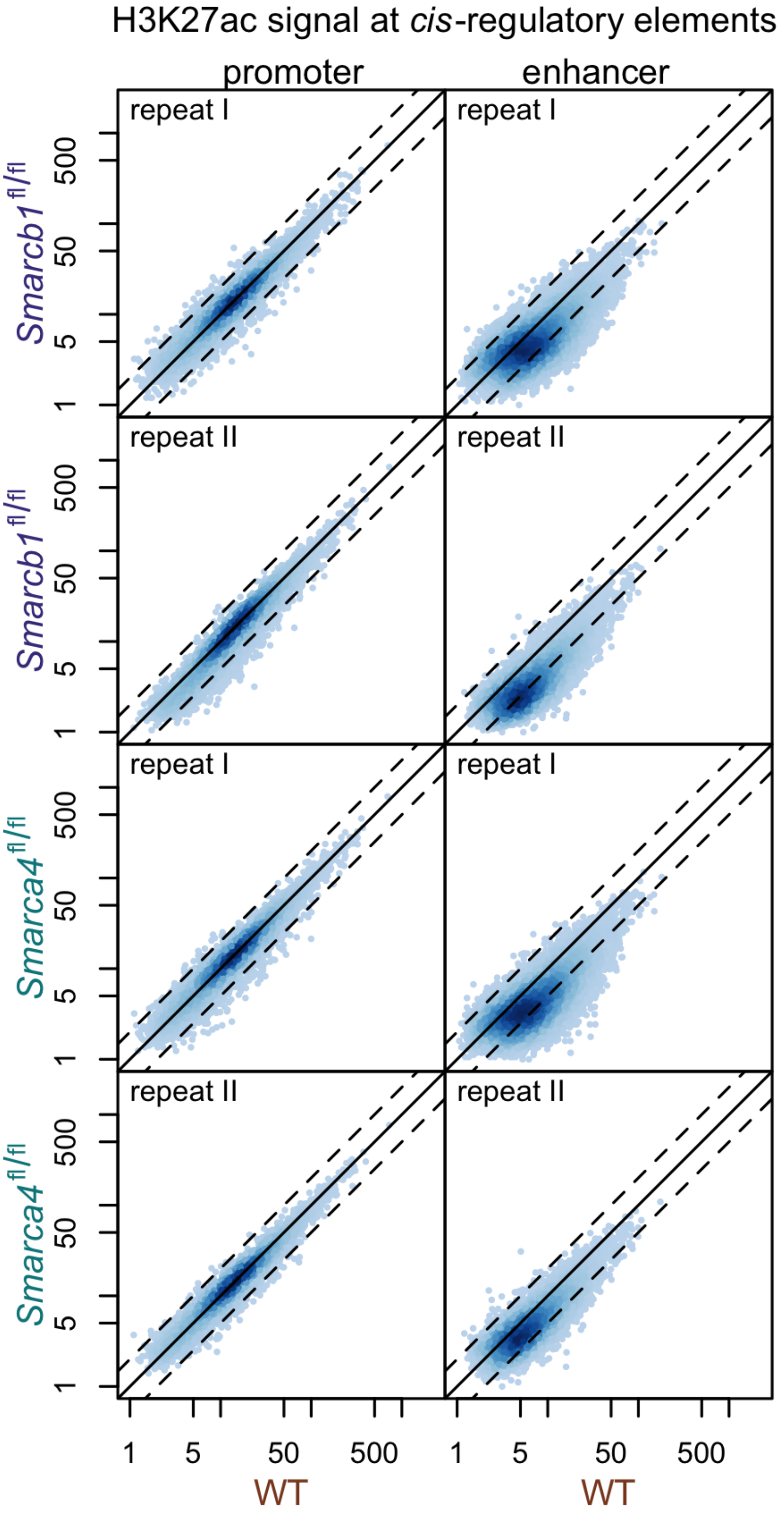

Supplementary Figure 7b: H3K27ac signal at promoters and enhancers in *Smarcb1*-deficient or *Smarca4*-deficient vs. wild-type MEFs, after applying a multiplicative correction to account for IP pull-down efficiency. Solid lines show y=x and the dashed lines show 2-fold difference. Compare to Supplementary Figure 5.

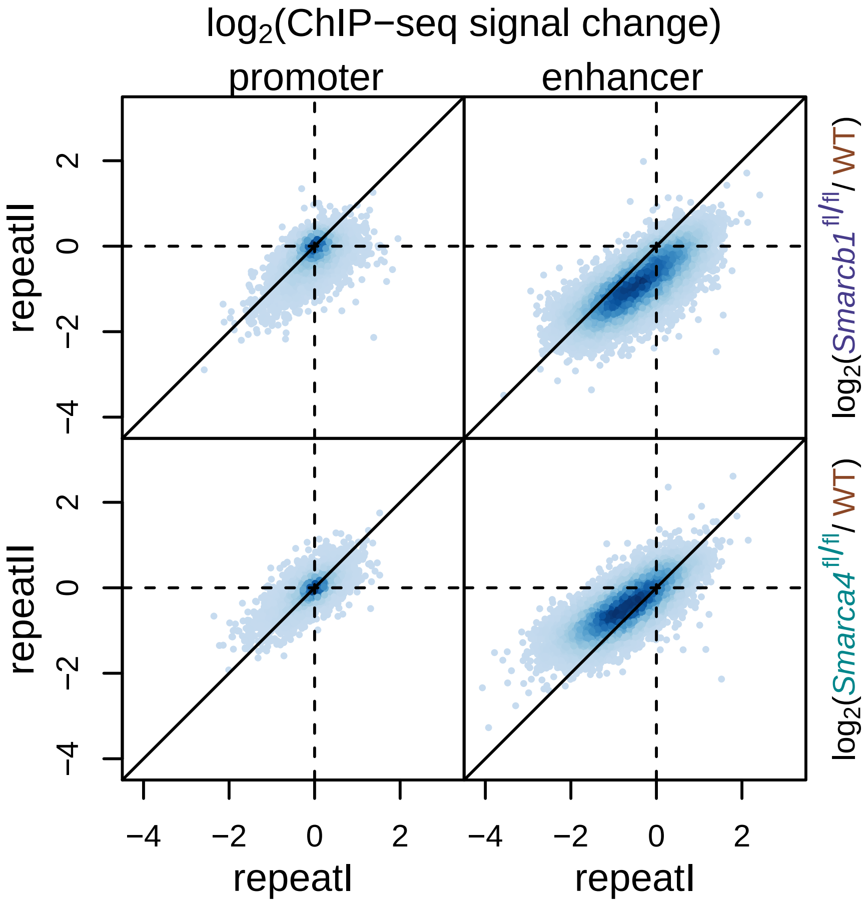

Supplementary Figure 7c: Log-fold change of H3K27ac signal at promoters and enhancers in *Smarca4*-deficient or *Smarcb1*-deficient relative to wild-type MEFs, showing repeat II vs repeat I, based on IP pull-down efficiency correction based normalization. Compare to Supplementary Figure 6.

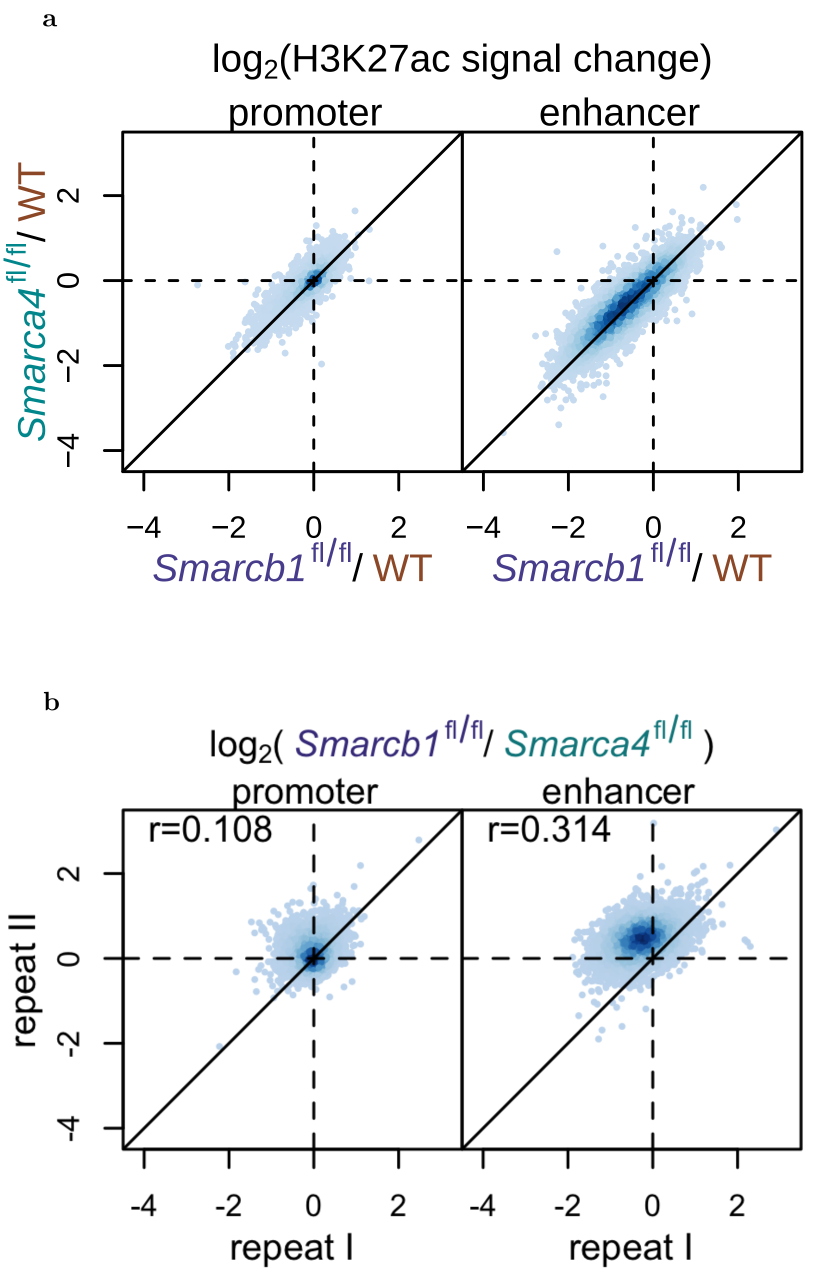

Supplementary Figure 8: a. Log-fold change of H3K27ac signal at promoters and enhancers in *Smarca4*-deficient vs. *Smarcb1*-deficient relative to wild-type MEFs. The changes observed for the two mutants are largely consistent. **b**. Log of the H3K27ac signal difference between *Smarcb1*-deficient and *Smarca4*-deficient MEFs. Two replicate experiment sets are plotted against each other. The difference at enhancers between the *Smarcb1*-deficient and *Smarca4*-deficient cells is small, but a reproducible difference is observed, as demonstrated by the positive correlation. This finding suggests that a specific sets of enhancers are affected differently by the loss of either subunit.

a

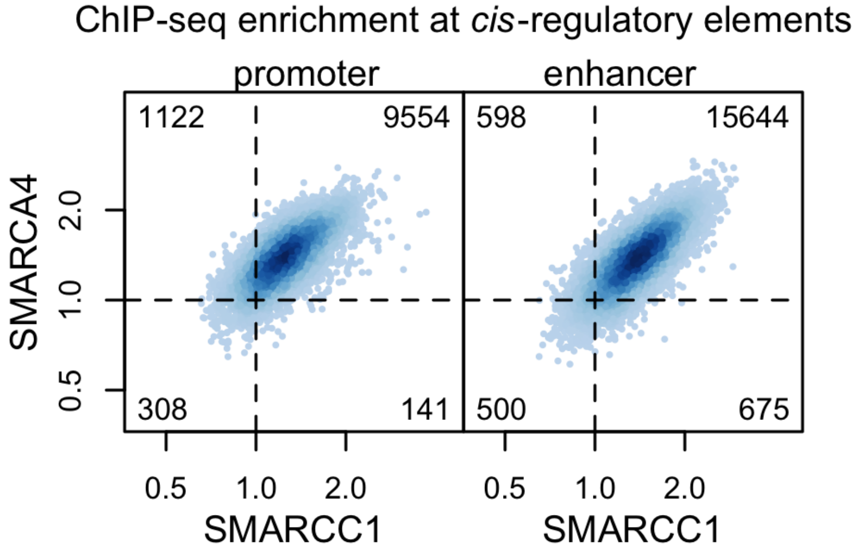

b

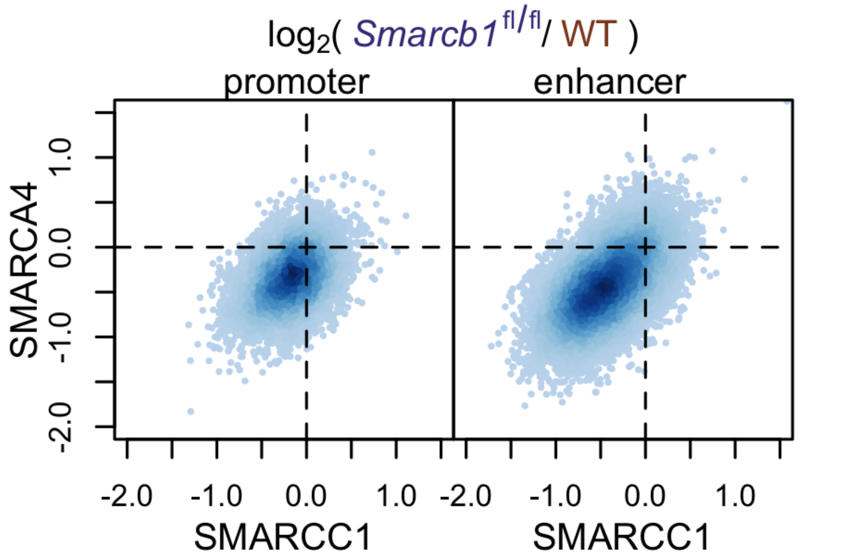

Supplementary Figure 9: **a**. SMARCA4 vs. SMARCC1 enrichment at promoters and enhancers in wild-type cells. The number of sites in each quadrant are displayed at the corners. The majority of *cis*-regulatory elements, defined by high H3K27ac signal, are found to be enriched for SWI/SNF binding. **b**. SMARCA4 vs. SMARCC1 fold-change at promoters and enhancers upon *Smarcb1* deletion. Most *cis*-regulatory elements lose SWI/SNF binding.

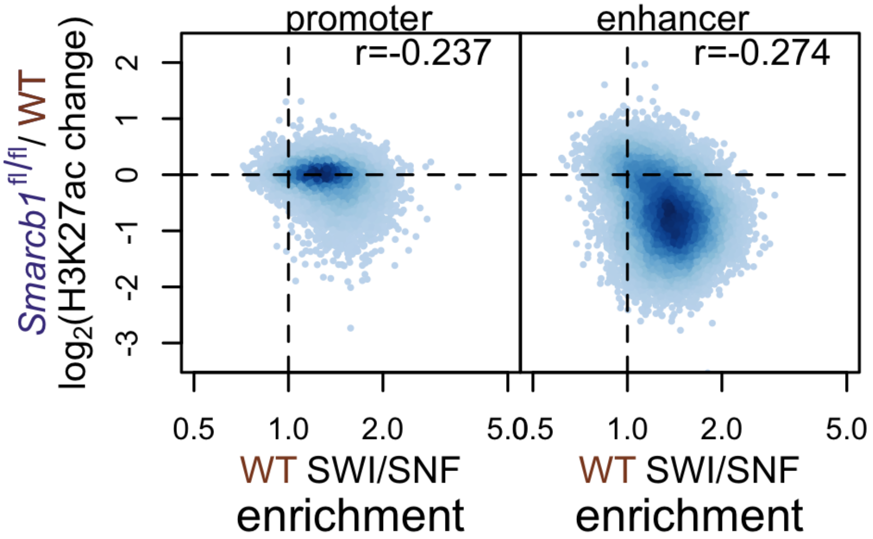

Supplementary Figure 10: The change in H3K27ac signal upon *Smarcb1* loss vs. SWI/SNF enrichment in wild-type MEFs. The enhancers with strongest SWI/SNF binding in wild-type are more likely to lose H3K27ac signal upon *Smarcb1* knockout.

a

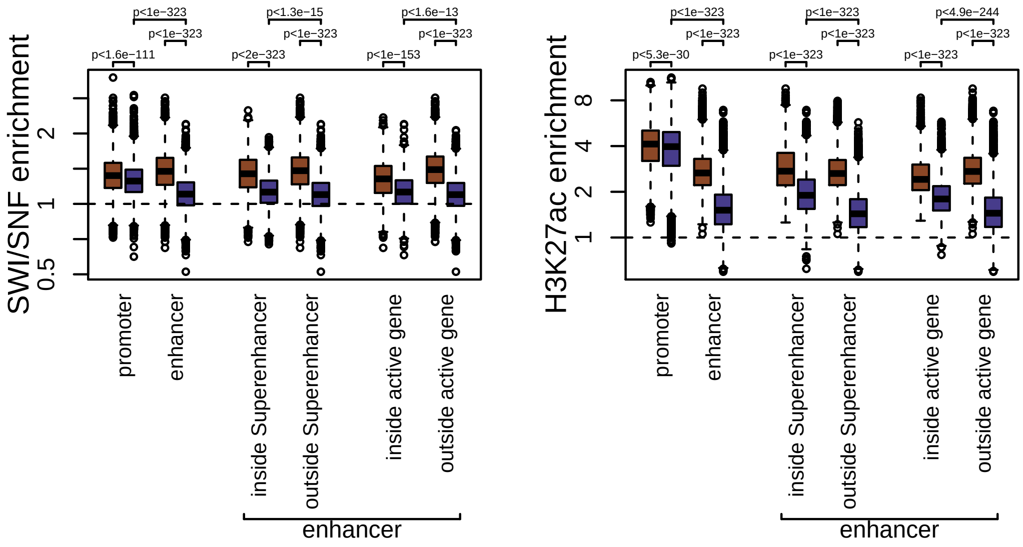

b

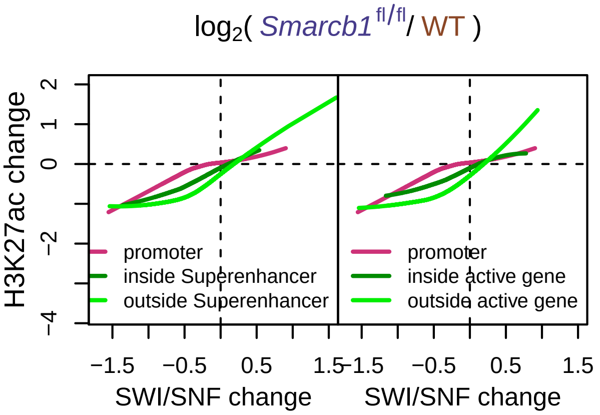

Supplementary Figure 11: a. Enrichment of SWI/SNF (average of SMARCA4 and SMARCC1, left) and H3K27ac (right) ChIP-seq enrichment relative to input, at different regulatory regions in wild-type and *Smarcb1*-deficient MEFs. **b.** The change in H3K27ac vs. SWI/SNF signal upon *Smarcb1* loss. Loess curves for different *cis*-regulatory regions are shown (promoter, inside or outside superenhancers, inside or outside PolII enriched transcribed regions).

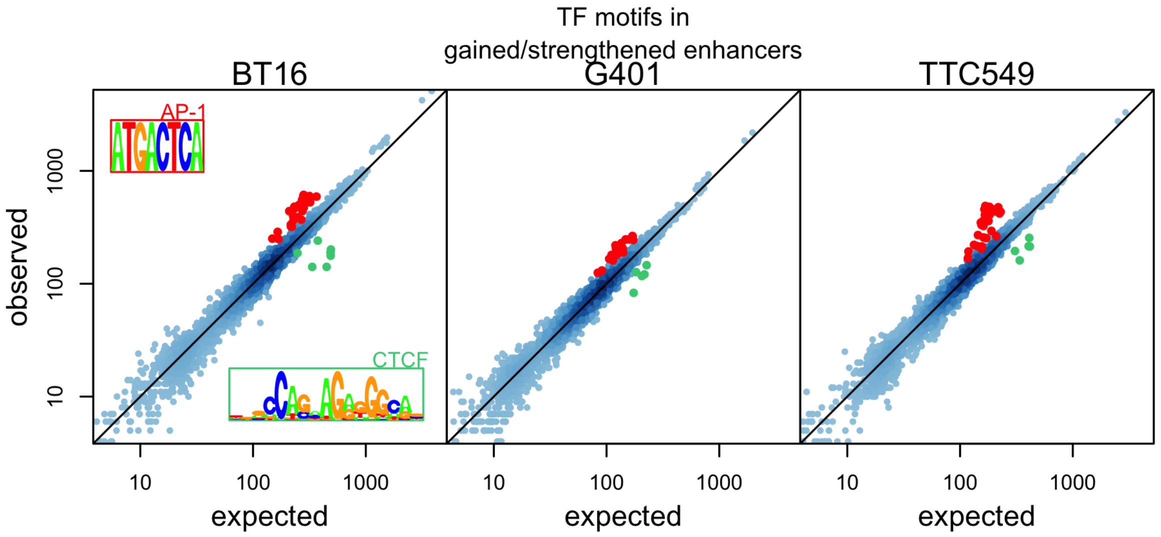

Supplementary Figure 12: BT16, G401, and TTC549 are three *Smarcb1*-deficient rhabdoid tumor cell lines. Re-expression of *Smarcb1* results in activation of a set of enhancers in these cells. This figure shows the number of transcription factor motif matches within gained enhancers relative to an expectation based on numbers in stable enhancers. The strongest enrichment and depletion is seen for motifs similar to AP-1 and CTCF recognition elements respectively.

Fig 1a

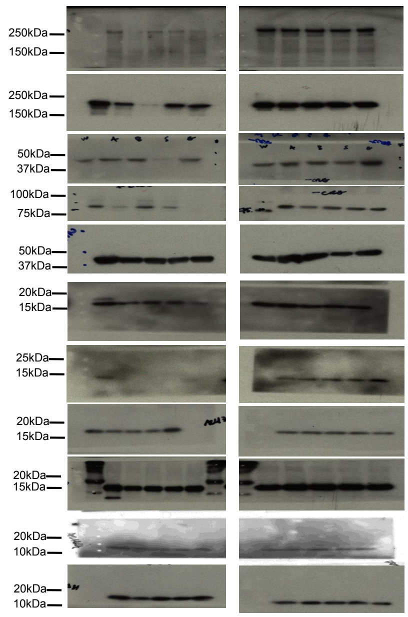

Fig 3a

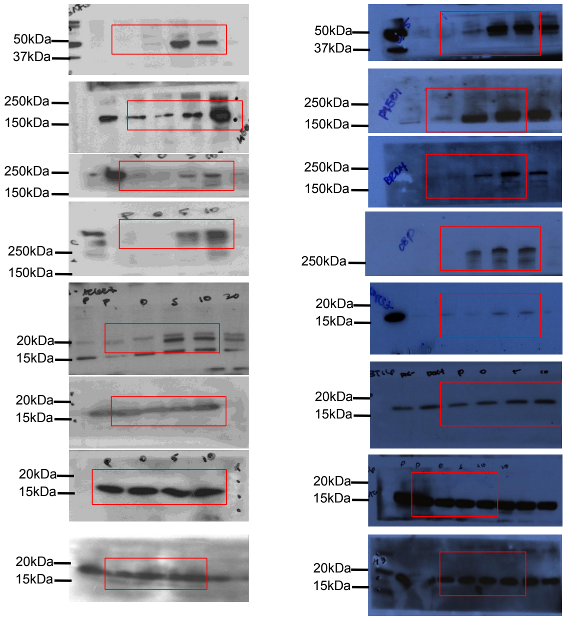

Fig 3b

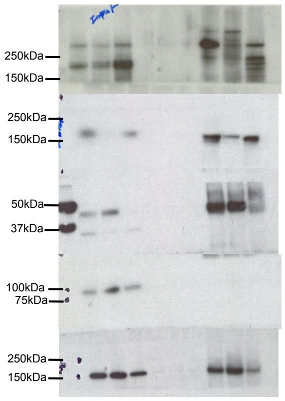

Fig 3c

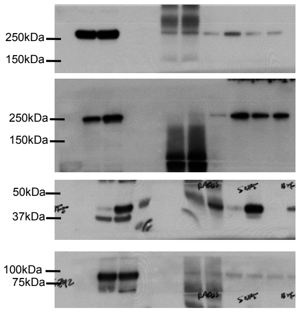

Fig 3d

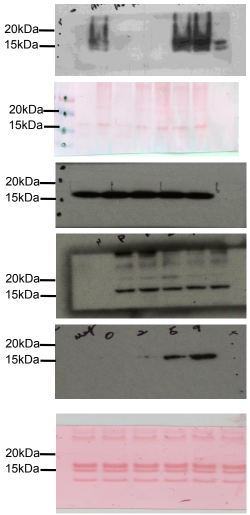

Sup Fig 1

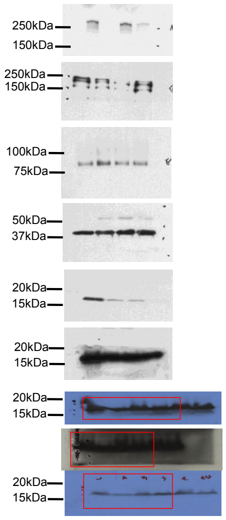

Supplementary Figure 13: Uncropped versions of the Western Blots are presented in the same order as in the figures.
